# Depression and its association with psychological factors among adolescents living with HIV in Southwestern Nigeria

**DOI:** 10.1186/s12888-023-04912-8

**Published:** 2023-07-24

**Authors:** Lawal RO, Adesanmi Akinsulore, Oginni OA, Aloba OO, Mosaku SK, Akanmu AS

**Affiliations:** 1 Federal Medical Centre, Abeokuta, Ogun State Nigeria; 2 Obafemi Awolowo University, Ile-Ife, Osun State Nigeria; 3 University of Lagos, Lagos, Lagos State Nigeria

## Abstract

**Objective:**

The aim was to determine the prevalence of depressive disorder and associated psychological factors among adolescents living with HIV/AIDS.

**Methods:**

One hundred and five adolescents with HIV were assessed for self-esteem, internalized HIV stigma and diagnosed of depressive disorder. Chi square and t tests were used to test associations and hierarchical logistic regression used to identify independent risk factors for depression.

**Results:**

The mean age of participants was 16.5 (± 1.97) years and 55.2% were males. Mean stigma scores were significantly higher in those with depressive disorder (16.53 ± 3.85) when compared with those without (13.42 ± 3.464) and this difference was statistically significant (t = 3.17, p = 0.01). The mean self-esteem scores were significantly lower in participants with depressive disorder (17.53 ± 4.69) compared to those without depressive disorder (19.71 ± 3.86), though not significant (t = 1.96, p = 0.053). Depressive disorder was further significantly associated with female sex, being in romantic relationship, decline in work output due to HIV and HIV stigma. Independent risk factors were sex and HIV stigma.

**Conclusion:**

Depressive disorder is common among adolescents living with HIV infection in Nigeria. The association between HIV stigma and depression, thus suggests the need for prevention strategies targeting the impacts of HIV infection among adolescents.

**Supplementary Information:**

The online version contains supplementary material available at 10.1186/s12888-023-04912-8.

## Introduction

Over two million adolescents in Sub Saharan Africa are infected with Human immunodeficiency virus (HIV)[1]and about 50% of new HIV infections occur in young people between the ages of 15 and 24 years [3]. Approximately 170 adolescents aged 15–19 years becomes infected with HIV everyday [2] indicating a high vulnerability of this group to HIV infection. An adolescent as defined by WHO, has someone between the ages of 10-19years [3].

Depression is the most prevalent psychiatric symptom in adolescents living with HIV/AIDS, lifetime prevalence rate ranges between 18% and 81% [4]. Depression as a disorder is poorly recognised and misdiagnosed among adolescents [5, 6]. A report by Rinke et al. showed 60% of adolescents were missed for diagnosis of depression [7]. Depression, if not recognised can increase adolescent mortality and adversely impact on engaging in different pursuits of life that will make life meaningful in future. The severity and outcomes of depressive disorder is worse among adolescents infected with HIV, thus making life more miserable for these adolescents.

Depressive symptoms in this vulnerable population could also be associated with other psychological factors such as personality strengths, resilience and coping strategies; worsening health outcomes such as poor linkage to care, poor medication adherence, risky behaviours, poor quality of life and poorer virological response to treatment.[8].

Depression among them may be due to some specific psychological factors such as decreased self-esteem and increased self-stigma resulting from the daily discrimination they experience or perceived [8].

Self-stigma and low self-esteem are major predictors to depressive illness. There is reluctance to disclose HIV status to others with resultant social isolation, self-hatred and disruptions in normal social relationships. This is also accompanied with decreased access to and retention in care, and poorer adherence to antiretroviral therapy.[3, 6, 7].

Despite the significance of depression in adolescents living with HIV, to my knowledge, no published studies have investigated the psychological associations of depression among adolescents in Nigeria. Kehinde et al. compared the prevalence of depression and associated clinical variables (orphanhood, academic failure and hospitalization) among children and adolescents with HIV and healthy controls [10]. Sale et al. looked at the prevalence of depression among youth aged 15–25 years and found stage of the disease, level of CD-4+, inability to afford medication, unemployment, lack of social support, inability to tolerate combination antiretroviral therapy (cART) to be associated with depression [11].

The present study therefore aimed to examine the psychological associations of depressive disorder, specifically self-stigma and self-esteem among adolescents. This knowledge will inform holistic interventions targeting these psychological associations and ultimately improving the physical and psychological care for these vulnerable adolescents.

## Methods

### Location of the study

This multicentre study was carried out at the Adolescent HIV clinics at Obafemi Awolowo University Teaching Hospitals Complex (OAUTHC) in Ile-Ife and Lagos University Teaching Hospital (LUTH) in Lagos. A pilot was carried out at the University College Hospital (UCH) Ibadan. The centres were located in South-Western states of Nigeria; Osun, Lagos and Oyo states respectively. These centres provide comprehensive health care for adolescents with HIV in their state and also to neighbouring states around them.

### Participants

One hundred and five adolescents aged 10–19 years were recruited consecutively at the HIV clinics over a period of 4 months when they presented for their outpatient clinics and cART prescriptions. Participants were those who had been diagnosed with HIV for at least one year so that they will have become fully aware of their HIV statuses, the psychosocial effects, associated stigma and self-esteem. Ethical clearances were obtained from the Ethics and Research Committee in OAUTHC (Protocol number ERC/2018/02/11) and Health Research Ethical Committee in LUTH (Protocol number HREC/APP/2379). Assent was obtained from the participants and written informed consent was obtained from parents or guardians.

### Measures

Sociodemographic questionnaires were used to assess socio-demographic characteristics of the participants. This included age, gender, ethnicity, level of study and peer relationships, parent marital status, parent employment status, and their highest educational attainment.

Illness-related variables such as duration of diagnosis of HIV infection, types and duration of antiretroviral therapy, side effects of antiretroviral therapy were obtained. CD-4 cell count at onset of diagnosis, most recent CD-4 + cell count, HIV status of parent, sibling and primary care giver and loss of family member to HIV/AIDS were also obtained from the patient and case file.

The internalized AIDS-related stigma scale is a 6-item self-report instrument developed by Kalichman et al. [12] to measure internalized AIDS-related stigma among people living with HIV/AIDs. The items are rated on a 4-point Likert scale ranging from 1 (Strongly disagree) to 4 (Strongly agree). Cronbach’s alpha in the present study was 0.75 and range of scores obtainable was 6–24 with higher scores indicating higher levels of internalized AIDS-related stigma.

Rosenberg self-esteem scale was developed by Rosenberg to measure self-esteem. It is a ten-item measure that is scored on a 4-point Likert scale ranging from 1 = strongly disagree to 4 = strongly agree. Five of the items [2, 5, 6, 8, 9] are negatively worded and reversely scored while the other five are positively worded [13]. Cronbach alpha in the present study was 0.86 and total obtainable scores ranged from 10 to 40, with higher scores indicating higher self esteem.

The Mini International Neuropsychiatry Inventory for Kids (MINI-KID) is the paediatric version of the Mini International Neuropsychiatry Inventory (MINI) designed by psychiatrists in Europe and U.S.A for use among children and adolescents. It is a brief structured diagnostic interview for Axis 1 psychiatric disorders in DSM-IV and ICD-10 [14]. These psychiatric disorders are represented in modules from A-X. The depression module was used in this study.

### Procedure

Participants who consented and were selected on the basis of the inclusion criteria on each clinic day were asked to complete the study measures within designated confidential areas of the clinics on each clinic day. Thereafter, all the study participants were interviewed by the researcher using the module on depressive disorder of MINI-KID diagnostic instrument. Participants were first administered the screening questions, which were components of the MINI-KID depression diagnostic instrument. All those who screened positive only for depressive disorder were administered the full diagnostic algorithm according to the responses of the participants. This process was repeated at each clinic until the desired sample size was reached, this was achieved within 4 months.

### Data analysis

The data collected was analysed using the IBM-SPSS software version 21 (IBM Corp., Armonk, NY USA). Means and proportions were used to describe socio-demographic and clinical variables. The associations between the outcome variables (depressive disorder) and independent variables (socio-demographic factors, family, HIV-related and psychological variables) were determined using relevant inferential statistics such as Chi-square and t-test as appropriate. Univariate binary logistic regression was used to determine variables that were associated with the diagnosis of depressive disorder. Both tests were two-tailed and the level of statistical significance was set at *p* < 0.05. Hierarchical logistic regression was further used to explore the multivariate relationship between depression and variables significantly associated with it at the bivariate level. Sociodemographic predictors were included in the first model while psychological predictors were further included in the second model.

## Results

### Socio demographic and illness related characteristics

The mean age of participants was 16.5 (± 1.97) years (Table 1), with over 90% of the participants in mid and late adolescence. There were more male participants (55.2%) and most of the participants (84.8%) were students. About two-third of the participants (62.9%) reported contracting HIV at birth during delivery. The mean age of diagnosis of HIV was 8.07 (± 4.5) years. Most of the participants (93.3%) were in WHO stage 1 classification of HIV. The mean CD-4 + cell count at diagnosis was 538.9 (± 475.00) and the mean of the most recent CD-4 + counts was 641.8 (± 493.09). The prevalence of depressive disorder was 14.3%. The mean score of HIV internalized stigma was 13.87 (± 3.67) and that of self-esteem was 19.4 (± 4.03).

**Table 1:** Socio-demographic, illness related and psychological characteristics of Participants

Variable		Frequency (n = 105)	Percentage (%)
Age group (in years)	10–13	9	8.6
	14–16	43	41.0
	17–19	53	50.4
Sex	Female	47	44.8
	Male	58	55.2
Occupation	Student	89	84.8
	Apprenticeship	16	15.2
Living situation	Live with Parents	78	74.3
	Live with others^#^	27	25.7
Romantic relationship	Yes	22	21.0
	No	83	79.0
Comorbidity	Yes	3	2.9
	No	102	97.1
Route of transmission	Through delivery Medical treatment Sharing sharp object Not aware of route	66 11 9 19	62.9 10.5 8.6 18.0
Age diagnosed (in years)	≤ 4 5–9 10–14 15–19	26 38 28 13	24.7 36.2 26.7 24
Work/Studies decline due to HIV	Yes No	24 81	22.9 77.1
Death of family member due to HIV	Yes No/not aware	25 80	24.7 75.3
Stage of HIV/AIDS	1 2 3 4	96 2 4 1	
Mean CD4 count	At diagnosis Most recent	**Mean** 538.9 641.8	
Psychological factors (mean and SD)	Self esteem HIV internalized self stigma	19.40 (4.03) 13.87(3.67)	

*Hausa, Efik and Ijaw # extended family, employer and pastor

### Bivariate association of depressive disorder with sociodemographic, illness related and psychological variables

There was a significant association between gender and depressive disorder (Table 2), in which a significantly higher proportion of female respondents was more depressed when compared to male respondents (χ^2^ = 8.79, *p* = 0.01). There was also a significant association between romantic relationship and depressive disorder, in which a higher proportion of participants who were in romantic relationships were more depressed when compared to respondents who were not (χ^2^ = 6.99, *p* = 0.02). A significantly higher proportion of participants who reported decline in work output or studies were more depressed when compared to respondents with no decline in work output or studies (χ2 = 5.63, p = 0.04). Participants with depression reported significantly higher mean stigma scores (16.53 ± 3.85) when compared with those who were not depressed (13.42 ± 3.464, t = 3.17, p *= 0.01*). The mean scores of self-esteem of participants with depressive disorder (17.53 ± 4.69) was lower than those without depressive disorder (19.71 ± 3.86), this was not statistically significant (t = 1.96, p = 0.05).

**Table 2:** Association of depressive disorder with sociodemographic, illness related variables and other study measures

Depressive disorder			Statistics		
Variable	Yes (n = 15)	No (n = 90)	χ^2^ /t	df	*p- value*
	n (14.3%)	n (85.7%)			
Age group (years)					
10–13	3 (33.3)	6 (59.1)	2.97	*2*	*0.227*
14–16	5 (11.6)	38 (71.7)			
17–19	7 (13.2)	46 (70.4)			
Gender					
Male	3 (5.2)	55 (94.8)	8.79	*1*	***0.007*** ^***#***^
Female	12 (25.5)	35(74.8)			
Occupation					
Student	12(13.5)	77(86.5)	0.58	*1*	*0.868* ^*#*^
Apprenticeship	3(18.8)	13(81.3)			
Romantic relationship					
Yes	7 (45.5)	15 (54.5)	6.99	*1*	***0.021*** ^***#***^
No	8(9.6)	75 (90.4)			
Living with whom					
Parents Others	8 (10.4) 7(25.0)	69 (89.6) 21(75.0)	3.58	*1*	*0.092* ^*#*^
Route of transmission					
Through delivery	7(10.6)	59(89.4)	5.13	*3*	*0.162*
Medical treatment	3(27.3)	8(72.7)			
Sharing sharp object	3(33.3)	6(66.7)			
Not aware of route	2(10.5)	17(89.5)			
Age at HIV diagnosis(years)					
> 10	4(12.1)	29(87.9)	1.84	*1*	*0.898* ^*#*^
11–20	11(15.3)	61(84.7)			
Loss of family to HIV					
Yes	4(16.0)	21(84.0)	0.08	*1*	*1.000* ^*#*^
No	11(13.8)	69(86.3)			
Stage of HIV					
1	13(13.3)	85(86.7)	1.25	*1*	*0.576* ^*#*^
II-IV	2(28.6)	5(71.4)			
Work/studies decline due to HIV					
Yes	7(29.2)	17(70.8)	5.63	1	0.041^#^
No	8(9.9)	73(90.1)			
Stigma score	16.3 (3.8)	13.4 (3.4)	3.17		***0.010***
Rosenberg Self-esteem score	17.53 (4.6)	19.7 (3.8)	1.96		*0.053*

### Multivariate analysis of predictors of depressive disorder

Table 3 shows the multivariate analysis of predictors of depressive disorder. These variables included gender, involvement in a romantic relationship, decline in work due to HIV and HIV stigma. Gender, being in a romantic relationship, and decline in work output were included in the first model and jointly explained 24% of the variance in depressive disorder. In the first model, gender was the only independent socio-demographic predictor of depression whereby male participants were 80% less likely to be depressed compared to female participants (*p* = 0.04, 95%CI 0.05–0.09). HIV stigma was included in the second model, and remained an independent predictor of depression (p = 0.02, OR = 1.30, CI 1.05–1.63) and explained 11% of the variance to depression. Supplementary analysis was conducted in which variables associated with depressive disorder at *p* < 0.1 in bivariate analyses were included (‘who the participants are living with’ and self-esteem). Self-esteem further explained 2.3% of the variance in depression. However, it was not an independent predictor of depressive disorder.

**Table 3:** Hierarchical regression of variables significantly associated with depressive disorder

	Model 1				Model 2			
	OR	95% CI		*p*-value	OR	95% CI		*p*-value
**Sociodemographic variables**								
Gender (ref – female)								
	**0.2**	**0.054**	**0.904**	**0.036**	**0.4**	**0.042**	**0.819**	**0.026**
Romantic relationship	0.4	0.110	1.440	0.160	0.5	0.114	1.866	0.217
Living status	3.0							
Decline in work/school due to HIV	0.3	0.094	1.16	0.74	0.3	0.086-	1.284	0.110
**Psychological variables**								
Stigma score					**1.3**	**1.053**	**1.629**	**0.015**
NR^2^	0.31				0.35			
ΔNR^2^	0.31				0.11			
*p*-value	0.001				0.005			

## Discussion

Most of the participants in this study were in the mid to late adolescent with a mean age of 16.5 years SD (1.97) and the reason could be because of the delay in transitioning from paediatric HIV clinic, as most of the participants were prenatally infected with HIV.Two-fifths of the respondents (21%) disclosed they were in a romantic relationship, which is also similar to the 25% reported in a study conducted in Burkina Faso [15] but lower than the 33% and 85% observed in Ugandan [16] and South African [17] studies respectively. This disparity may be explained by cultural differences in acknowledging intimate relationships by adolescents in south western Nigeria.

None of the participants reported getting infected through sexual route. This could be related to the stigma of associating self with sexual misconduct which is the common myth attributed to the cause of HIV infection.

The prevalence of 14.3% for depressive disorder among the study participants was consistent with similar studies [18, 19]. Slightly higher prevalence were 17.8% and 20% from studies among samples with similar socio-demographic profiles to the sample in the present study [20, 21]. However, other studies done in Nigeria, Kenya and Uganda among adolescents and youth with HIV infection found higher prevalence rates of 39%, 52% and 46% respectively [11, 22–24]. This could be due to methodological differences, while previous studies used self-reported instruments to estimate symptoms of depression, the present study employed a diagnostic instrument to make definitive diagnosis of depressive illness.

A possible explanation for the association between HIV self-stigma and depressive disorder in this study could be the societal view of immorality been associated with HIV infection and the view of labelling HIV infection as a death sentence [25]. Persistent view of hopelessness, dejection and negative self-view and poor self-worth could have accounted for the increased risk to depression in them.

Self-esteem was also shown to be associated with depression among the participants and this is consistent with findings from a study done by Okwaraji et al. [26] among adult HIV patients. Negative self-image, which was associated with self-esteem as found in supplementary analyses in the present study, could have been responsible in this age group. However, while HIV self-stigma remained an independent predictor of depression among the participants, the effects of self-esteem remained non-significant in bivariate analyses. This suggests that HIV-self-stigma rather than self-esteem confers a stronger risk for depression, however, this needs to be tested using a prospective design.

## Conclusion

This study showed that one in seven adolescents living with HIV in Southwestern Nigeria has depressive disorder. The sociodemographic and psychological associations with depressive disorder were also highlighted. This study also supports previous findings in literature and gives further evidence of the need to provide a multifaceted approach to care in adolescents with HIV infection.

### Limitations

The not so large sample size limits the capacity to detect significant associations. Also, the inability to determine the temporal relationship between depressive disorder and HIV were not ascertained, therefore limiting inferences regarding cause and effect.

### Recommendations

HIV stigma and low self-esteem can impact on indices of well-being, including quality of life; and health care access, leading to negative consequences and poor health outcomes. Services to address stigma and other mental health morbidity are low in our HIV adolescent clinics and thus necessitating increased clinical focus and research. Also, if not properly addressed it could limit the occupational opportunities of these adolescents as they may not be engaged productively causing a future loss in revenue to the country.

## Electronic supplementary material

Below is the link to the electronic supplementary material.

Supplementary Material 1: Certificates of evidences for ethical, consent and assent forms

## Data Availability

It will be made available on request by the corresponding author when needed.
