# Supplementary material for: Depression and its association with psychological factors among adolescents living with HIV in Southwestern Nigeria
Source: BMC Psychiatry. 2023 Jul 24;23:531. doi: 10.1186/s12888-023-04912-8 (PMC10367253; doi:10.1186/s12888-023-04912-8)
Supplement: Supplementary file 1 — Supplementary Material 1: Certificates of evidences for ethical, consent and assent forms [file 12888_2023_4912_MOESM1_ESM.docx]

**APPENDIX 1**
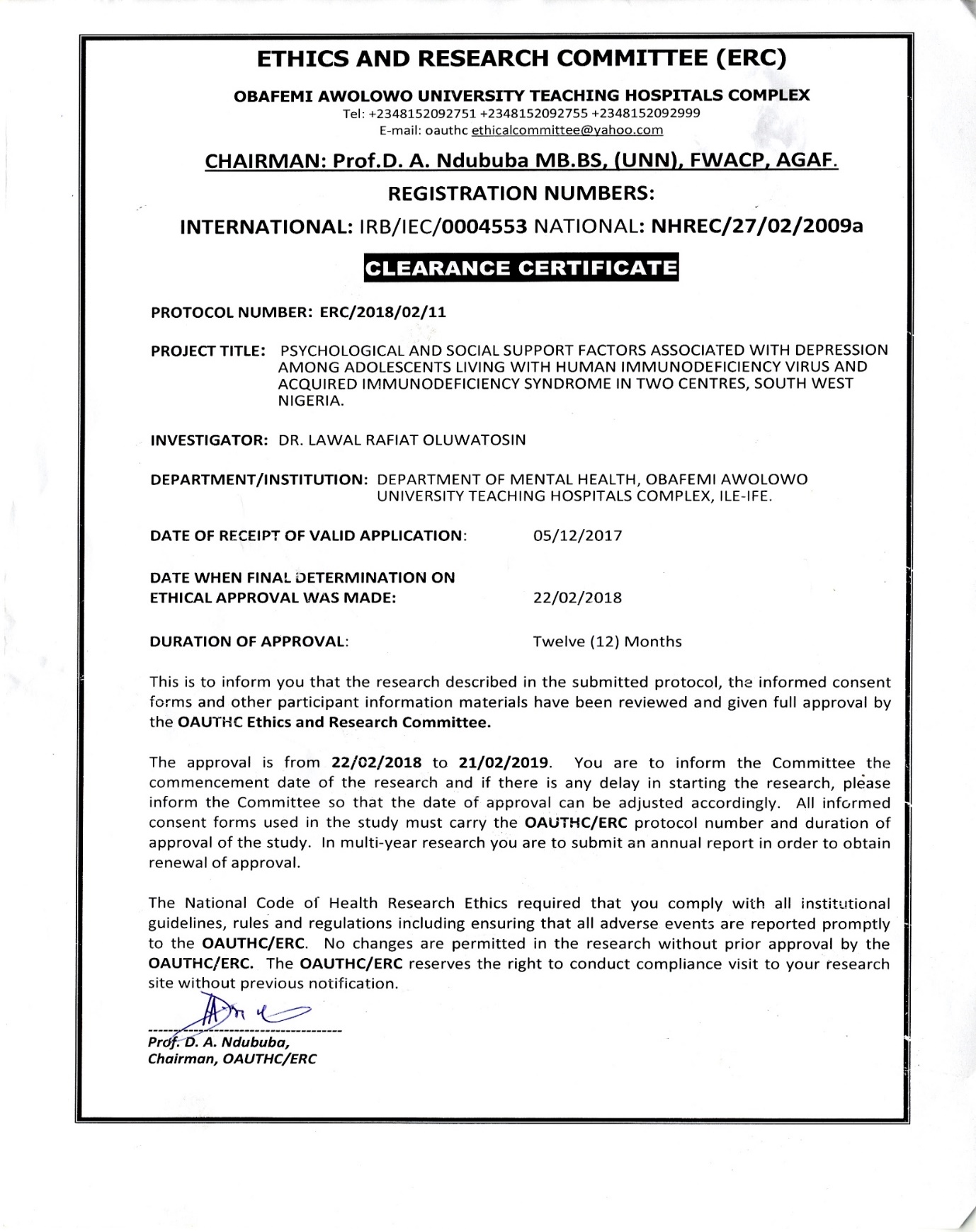
**APPENDIX 2**
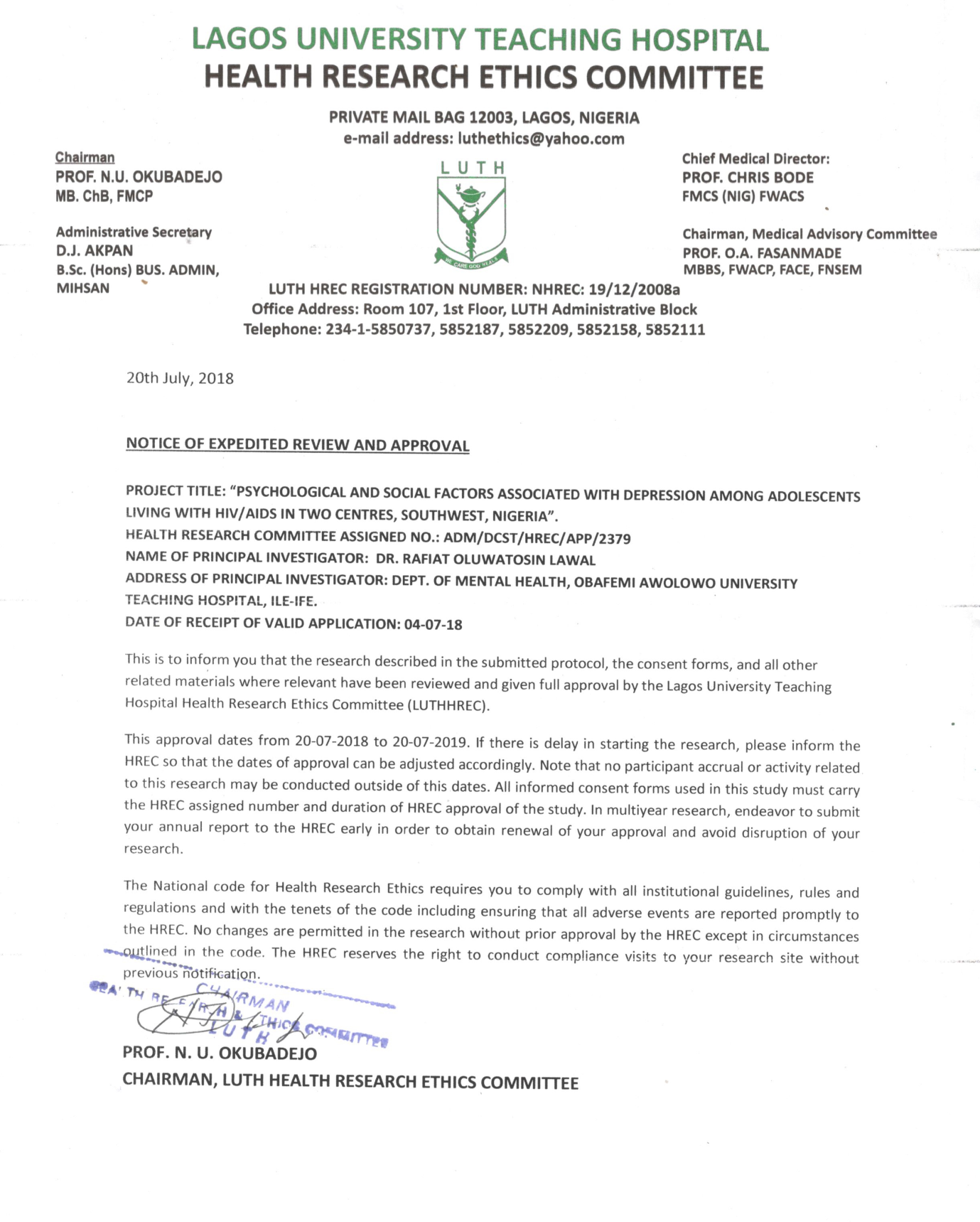


**APPENDIX 3**

**CONSENT FORM**

***OBAFEMI AWOLOWO UNIVERSITY TEACHING HOSPITALS COMPLEX, ILE-IFE.***

**PSYCHOLOGICAL AND SOCIAL SUPPORT FACTORS ASSOCIATED WITH DEPRESSION AMONG ADOLESCENTS LIVING WITH HIV/AIDS IN TWO CENTRES, SOUTHWEST NIGERIA**

***Subject’s Agreement/Consent Form:***

I have read the information provided in the Subject Information Sheet, or it has been read to me.

I have had the opportunity to ask questions about the research and all questions I have asked have been answered to my satisfaction. I consent voluntarily to participate in this study and understand that **(my record will be reviewed and I will be administered questionaires**) and I have the right to withdraw from the study at any time.

|  |
| --- |

|  |
| --- |

**Yes No No**

**------------------------------------------------------------------------------------------------------**

Signature/Thumb print of Research Respondent. Date:

If participants cannot read: Signature of Mother or Legal Guardian.

Signature/thumb print of Person Obtaining Consent. Date:

Name of witness Signature Date

Printed Name of Person Obtaining Consent.

***APPENDIX 4***

***OBAFEMI AWOLOWO UNIVERSITY TEACHING HOSPITALS COMPLEX, ILE-IFE.***

**ASSENT FORM**

**PSYCHOSOCIAL FACTORS ASSOCIATED WITH DEPRESSION AMONG ADOLESCENTS LIVING WITH HIV/AIDS IN SOUTHWESTERN NIGERIA**

***Parent/Guardian Consent Form:***

I have read the information provided in the Subject information Sheet, or it has been read to me.

I have had the opportunity to ask questions about it and any questions I have asked have been answered to my satisfaction. I consent voluntarily to allow my child/ward participate in this study and understand that ***(he/she case note will be reviewed and questionaires be administered)*** he/she has the right to withdraw from the study at any time.

|  |
| --- |

|  |
| --- |

**Yes No**

**------------------------------------------------------------------------------------------------------**

Signature/Thumb print of Research Respondent’s Mother or Legal Guardian. Date:

Signature/thumb print of Person Obtaining Consent Date:

Name of witness& Signature

Printed Name of Person Obtaining Consent.

***Child/Ward Agreement (Verbal)***

Although I am not yet 18 years old but I have had the opportunity to ask questions about this research and any questions I have asked have been answered to my satisfaction. I consent voluntarily to participate in this study and understand that I have the right to withdraw from the study at any time.
